# Transcription of the Tumor Suppressor Genes *p53* and *RB* in Lymphocytes from Patients with Chronic Kidney Disease: Evidence of Molecular Senescence?

**DOI:** 10.1155/2012/154397

**Published:** 2012-09-23

**Authors:** Vasileios Kordinas, Chryssoula Nicolaou, George Tsirpanlis, Anastasios Ioannidis, Sotiris Bersimis, Nikos Sabanis, Eleni Fragou, Konstantina Tsiolaki, Stylianos Chatzipanagiotou

**Affiliations:** ^1^Department of Medical Biopathology and Clinical Microbiology, Aeginition Hospital, Medical School, University of Athens, 115 28 Athens, Greece; ^2^Department of Nephrology, General Hospital of Athens, “G. Gennimatas”, 115 27 Athens, Greece; ^3^Department of Nursing, Sparta General Hospital Building Complex, Faculty of Human Movement and Quality of Life Sciences, University of Peloponnese, 23 100 Sparta, Greece; ^4^Department of Statistics and Insurance Science, University of Piraeus, 185 34 Piraeus, Greece

## Abstract

Patients suffering from renal failure exhibit an impaired immune system function. We wanted to investigate the transcription of the tumor suppressor genes *p53* and *RB* to record, if these cells could be stimulated *in vitro* in order to divide, after the addition of antigenic and inflammatory factors. This expression was measured by real-time PCR in peripheral blood mononuclear cells (PBMCs) from three different groups: ten healthy individuals, ten patients with chronic kidney disease (CKD), and ten dialysis patients with end stage renal disease (ESRD). The transcription rate of these genes was also measured after the cultivation of PBMCs under four different conditions: just with the culture medium, with lipopolysaccharide (LPS), with C-reactive protein (CRP), and with lipoxin A_4_ (LXA_4_)-LPS. Our results show that in most cases after the cultivation with additives, the transcription levels were higher in dialysis patients compared to those of the other two groups. Our findings serve as indications of cellular senescence on a molecular level, while it seems that these cells are less easily stimulated *in vitro* in order to duplicate.

## 1. Introduction

Patients with CKD and ESRD suffer from an impaired immune response. According to previous studies, this population is strongly associated with chronic inflammation. This continuous inflammatory state predisposes patients to cardiovascular diseases and acute infectious complications, two of the main causes of death in this population. Despite proper pharmaceutical therapy and/or adequate hemodialysis, mortality rates in these groups of patients are much higher than in the general population and it seems that the dysfunction of the immune system plays a key role in the pathogenesis of many disorders associated with renal failure [1–3].

The tumor suppressor genes *p53* and *RB* are possibly the two most known and well-studied genes in this particular category. These genes control the G1→S phase of the cell cycle transition and their downregulation is absolutely necessary in order for the cell to duplicate [4–6]. Whenever for example there is a DNA damage or genome instability, complex molecular networks mediated by these two genes halt the cell cycle in order for the DNA to be repaired before duplication [4, 6]. Therefore p53 has been called “the guardian of the genome” [4] and these two genes have been the subject of extensive investigation worldwide. Apart from controlling the cell cycle progression, *p53* has also the ability to induce apoptosis [4, 6]. Another important point of their function is that the vast majority of cancer cells exhibit either turned off, or mutated *p53* and *RB* genes [7]. 

In a previous study, we found that telomerase activity, an enzyme that provides an almost unlimited proliferation capacity when active, is decreased in peripheral blood mononuclear cells (PBMCs) from dialysis patients, when compared to healthy individuals [8]. Simultaneously, this activity was inversely correlated with serum TNF-*α* concentration in the population studied [9]. In the present study, immune cells belonging to renal patients were examined from a different perspective. Since telomerase, an enzyme that deals with cell division, is less active in renal failure, the transcription patterns of two very important genes implicated in cell division were determined. Thirty different individuals were chosen, ten from each of the following categories: healthy controls, CKD patients, and ESRD (dialysis) patients. PBMCs were purified and cultivated in four different conditions: (i) without the addition of any factor other than culture medium with antibiotics (plain culture), (ii) with the addition of lipopolysaccharide (LPS), (iii) in presence of C-reactive protein (CRP), and (iv) with the addition of LPS and Lipoxin A_4_ (LXA_4_). LPS or endotoxin is a molecule located at the cell wall of Gram negative bacteria and has the ability to promote a strong inflammatory response [10]. CRP is one of the most well-investigated acute phase proteins and its main role is to bind to phosphocholine expressed on the surface of dead or dying cells or bacteria, in order to activate the complement system and the phagocytosis by macrophages [11]. LXA_4_ is an anti-inflammatory agent derived from arachidonic acid [12].

Peripheral blood mononuclear cells are a diverse population consisting mainly of lymphocytes [13]. The aim of the study was to explore any possible differences in the transcription of both tumor suppressor genes before and after the various cultivation conditions, among the different study groups. The inflammation status of every individual included in the study was assessed by the determination of interleukin 6 (IL-6), IL-10, C-reactive protein (CRP), and TNF-*α* in blood serum.

## 2. Materials and Methods

### 2.1. Patients and Healthy Controls

The population studied consisted of three groups: ten healthy individuals (normal controls), ten chronic kidney disease (CKD) nondialysis patients, and ten dialysis patients with end stage renal disease (ESRD), subjected to hemodialysis several times in a week. Age, sex, GFR, and other relevant data of the three groups are shown in Table 1. An interview was conducted with every person before his/her admission to the study. Through this interview it was defined if that person was a smoker or not, while a blood glucose value of >110 mg/dL in three different measurements was used as the definition of diabetes. Hypertension was defined through a blood pressure mean value of >140/80 mmHg or antihypertensive treatment while in order to decide if a person suffered from atherosclerosis he/she had to have a history of angina, and/or vascular interventions, or a history of any cardiovascular event (e.g., past myocardial infarction). The New York Heart Association definitions were used for the assessment of heart failure in the population studied. The CKD group consisted of patients with a declined renal function and decreased GFR (GFR values from 15 to 60 mL/min, calculated with the Cockcroft-Gault formula) not needing dialysis, and the dialysis group comprises of patients with totally diminished kidney function depending on dialysis sessions several times a week. The mean value of the time in months that these patients had been subjected to dialysis sessions was 63.88 (St. Dev. 50.47). In four patients the dialysis filter was made out of cellulose, while in six patients a synthetic filter was used. In nine dialysis patients the vascular access was a fistula, and in one patient a graft. 

### 2.2. Blood Samples Collection

In all groups a 15 mL heparinized and a 5 mL nonheparinized blood samples were collected from each patient, for the preparation of PBMCs and serum samples, respectively. In dialyzed patients, blood samples were taken just before the onset of the midweek dialysis session.

### 2.3. Preparation of PBMCs

PBMCs were obtained by a Ficoll-Hypaque (Histopaque 1077, Sigma Aldrich, St Louis, Mo., USA) gradient density centrifugation (400 g for 30 min). The mononuclear layer was then collected and washed 3 times in PBS, and finally four aliquots containing 4∗10^6^ cells were transferred into Eppendorf tubes. These cells were immediately cultured in preprepared vials as described below.

### 2.4. CRP and Cytokines Assays

Blood serum was isolated by centrifugation of the 5 mL non-heparinized blood vial in 3200 rpm for 25 min. The quantitative determination of CRP was assayed by particle-enhanced immunonephelometry on a Behring Nephelometer II (Dade Behring, Deerfield, Illinois, USA). IL-6, IL-10 and TNF-*α* serum levels were quantified with a commercial sandwich enzyme immunoassay technique based on a monoclonal-polyclonal antibody pair (R&D systems, Minneapolis, Minn., USA). All reactions were carried out according to the manufacturer's instructions.

### 2.5. PBMC Cultures

The vials containing the medium with the necessary supplements for the cultures were preprepared in order for the cells to be placed into cultivation immediately after isolation. Fifteen mL polypropylene round-bottom tubes (Falcon, Becton Dickinson, Lincoln Park, N.J., USA) were used and each culture was adjusted to a concentration of 2∗10^6^ cells/mL in RPMI 1640 culture medium (Sigma Aldrich, St Louis, Mo., USA) supplemented with 1% heat-inactivated fetal bovine serum (Sigma Aldrich), 5.000 U/mL penicillin and 3.000 g/mL streptomycin. The four culture sets contained the following additives: (a) controls without any additive (plain cultures), (b) LPS 5 *μ*g/mL (Sigma-Aldrich, *Escherichia coli* 0111:B4), (c) human CRP 50 *μ*g/mL (USBiological, Swampscott, Massachusetts, USA), (d) LPS 5 *μ*g/mL and 10 nmol/L LXA_4_ [5(S),6(R)-Lipoxin *A*_4_, Cayman Chemical, Ann Arbor, Michigan, USA)]. LXA_4_ was added 30 minutes after the culture onset. The incubation lasted for 24 h at 37°C in a humidified atmosphere containing 5% CO_2_, and the Falcon tubes had loosened caps. Cells were collected by centrifugation at 3,000 g for 5 min at 8°C and stored at −80°C while the supernatant was discarded.

### 2.6. Real-Time PCR

RNA was purified from PBMCs with NucleoSpin 96 RNA kit (Macherey-Nagel's GmbH & Co. KG, Düren, Germany), according to the manufacturer's instructions. Reverse Transcription PCR was performed on RNA purified samples with Superscript III Reverse transcriptase (Invitrogen, Carlsbad, CA, USA) as described in the instructions' manual. 4∗10^6^ cells per sample were used for the RNA purification. From the isolated RNA, 11.5 *μ*L per sample were transcribed and the resulting cDNA was used in the real-time PCR procedure. Real-Time PCR was performed on SDS System 7300 (Applied Biosystems, Foster City, CA, USA). Assays-On-Demand 20x assay mix of primers and FAM dye-labeled TaqMan MGB probes (Applied Biosystems, Foster City, CA,USA) were employed to calculate the relative expression of *p53 *and *RB *(Assay ID: Hs00153349_m1 and Hs00153108_m1 resp.). Predeveloped GAPDH (Glyceraldehyde 3-phosphate dehydrogenase—a “housekeeping” gene) TaqMan assay reagent was also used for endogenous control. The cycling parameters and experimental procedures were those of the manufacturer. Each experiment was performed under the exact same circumstances and in duplicates in order to be able to correct any possible mistakes. To avoid any bias, RNA isolation and real time PCR experiments were performed with multiple samples simultaneously and the samples were later on categorized with respect to the group that each belonged to, after each reaction was completed. The Ct (cycle threshold) values obtained at the end of the procedure were transferred to REST software (Relative Expression Software Tool, v2.0.7, copyright 2008, Corbett Research Pty. Ltd.) for the relative expression to be calculated. Each sample was tested in duplicates. Since the relative quantification method was used for the assessment of the gene transcription rates, only pair wise comparisons could be made. These comparisons are shown in Table 2 with the differences in expression, the difference factor and the statistical significance. The term “difference factor” stays for the difference in gene expression between the two groups. Ct values calculated from the real time PCR experiments were analyzed by REST software with the first group in each pair being always considered as the “control” group while the second one is the group to be tested. The result of this comparison shows the difference in expression between two conditions. “Difference factor” states the increase or decrease of the expression in folds. If for example the result between two groups is a downregulation of *p53* by a 0.1 factor, then the second group exhibits a tenfold decrease in *p53* expression when compared to the first group.

### 2.7. Statistical Analysis

SPSS from IBM (Armonk, NY, USA) was the statistics software used. Serum inflammation markers were analyzed by the one way ANOVA test, while the statistical significance of the real-time PCR results was calculated by rest software that uses the hypothesis test through many randomizations techniques. 

## 3. Results

### 3.1. Inflammation

The mean values and the statistical analysis of the inflammation markers measured in each group are shown in Table 1. Only TNF-*α* was found to be significantly elevated in both patient groups as compared to the healthy controls. After statistical analysis (One-Way ANOVA tool), TNF-*α* seems to become elevated as the disease progresses. The Dialysis patients had higher concentrations than the CKD ones, while the lowest values were observed in the control group (*P* < 0.05).

### 3.2. Gene Transcription

**Figure 1:**
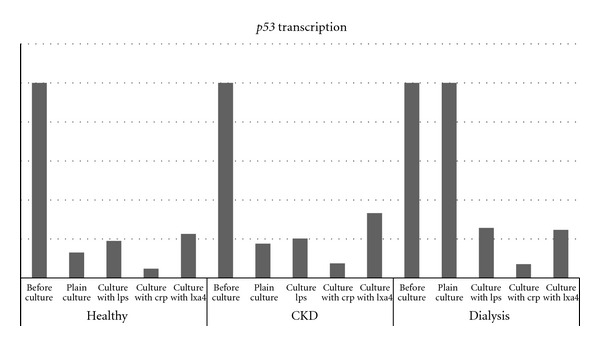
Expression status of *p53* in every condition studied. As the disease advances and especially in the dialysis group, the gene's transcription levels become higher in the corresponding cultures. The columns of this particular figure are derived from the pair wise comparisons depicted on Table 2.

**Figure 2:**
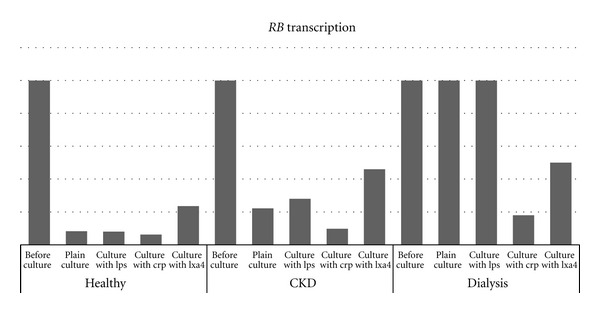
Expression pattern of *RB* in every possible group and condition. As the disease advances the gene's transcription levels become higher in the corresponding cultures. No matter the culture conditions *RB* is transcribed highly in dialysis patients. The columns of this particular figure are derived from the pair wise comparisons depicted on Table 2.

As stated before, for the assessment of the gene transcription rates, the relative quantification method was used and thus, only pair wise comparisons could be made. These comparisons are shown in Table 2. The expression status of both *p53* and *RB* was found to be in the same level in all three groups in the cells immediately after purification (before culture). After cultivation of PBMCs from control samples, *p53* demonstrated a decrease in expression by 5-fold or higher. The expression of *p53* in the CKD and the dialysis groups was less decreased than in the control group in the corresponding cultivations. This was more evident in the dialysis group where the cells in the plain cultures demonstrated the same *p53* expression as cells before culture. Similar results were obtained when the expression of *RB* was measured after the completion of each culture. With the progression of the disease, the gene demonstrated remarkably higher transcription rates in the corresponding cultures in a more obvious way than p53. In the dialysis group, the transcription status of *RB* was the same before cell culture as well as after the plain cultivation and after the LPS culture. Figures 1 and 2 illustrate the above results. These figures are divided by the group each sample belonged to and they are the outcome of the pair wise comparisons depicted in Table 2. As explained thoroughly in Section 2 due to the use of the relative quantification method, a certain gene's expression is calculated only through pair wise comparisons performed with a particular computer software (REST). This is why there are no numbers in Figures 1 and 2. The bars show the differences in expression between the different conditions and their height does not correspond to a certain numerical value. The graphs are just a visualization of Table 2.

## 4. Discussion

*RB* and *p53* are very important tumor suppressor genes controlling the cell cycle, being downregulated or inactivated in order for a cell to divide [14, 15]. In the present study, there were no differences in the transcription status of *RB* and *p53* in freshly isolated PMBCs (before culture) among the patients and the healthy controls. However, differences were observed after the completion of four different sets of cell cultures either with or without any additives. Both genes were highly downregulated in healthy controls independently of the culture conditions, whereas they were more actively transcribed in CKD patients and even more in the dialysis group under the respective culture conditions as in the healthy controls. It is very likely that lymphocytes can promptly respond to environmental changes, by regulating gene transcription in a way that the cell cycle is not inhibited in its progression. This is very important for the PMBCs when for example an infectious agent must be eliminated. A large percentage of renal patients suffer from a chronic inflammatory state and bear an impaired immune function [16–18]. Here we demonstrated that TNF-*α* serum concentration increases as the disease advances, with the dialysis patients exhibiting the highest TNF-*α* concentrations. In the same time, both patient groups and especially the dialysis group exhibited a limited capacity in regulating *p53* and *RB* expression *in vitro*. Both genes and mainly *RB* demonstrated high transcription levels *in vitro*, even when LPS or CRP were present. The transcription rates of these very important tumor suppressor genes are a very strong indication that lymphocytes from dialysis patients had become senescent, a fact that if true, prohibits these cells from dividing, even in the presence of toxic stimulants. 

In the culture containing LPS, 30 minutes after the onset, an anti-inflammatory agent, LXA_4_ was added. The question was as how a molecule that inhibits the progression of inflammation could influence the transcription of the two genes. In both, healthy and CKD lymphocytes, the LXA_4_ culture yielded higher transcription rates of *p53* and *RB* within the same group, whereas the opposite was observed in the dialysis group. In the presence of LPS alone, PBMCs from the first two groups downregulated *RB* and *p53*, probably because they recognized a threat in their environment, and they should become activated and proliferate in order to eliminate it. In the dialysis group, however, the LPS culture exhibited higher transcription rates for both genes, when compared to the LXA_4_ culture, showing that these immune cells cannot respond properly in the presence of a toxic factor, a sign of immune system dysfunction, as observed in the dialysis group. 

Levels of p53 in cells not undergoing any kind of stress are kept low through a continuous degradation procedure [19]. Here we demonstrated that *p53* exhibits the same transcription level in all three groups of the population studied. So if we make the assumption that healthy cells are unstressed cells, then every cell in the before culture condition exhibits the same, “unstressed” transcription level. Though the transcription “before culture” is stable the differences in the after culture experiments are vast. Although this is more evident in *RB'*s transcription, it can also be seen in *p53'*s case. One cannot easily ignore the fact that although healthy plain culture cells exhibit a decreased expression of *p53*, dialysis plain culture cells exhibit exactly the same level of expression. In other words, it is very likely that expression of *p53* becomes deregulated as the disease advances. Of course we are not in the position to know what exactly is going on in the protein level, but the evidence we present cannot be easily ignored. These results do not serve as strong evidence of our hypothesis but they clearly serve as hard indications. One cannot oversee easily the fact that dialysis cells exhibit high *p53* transcription levels even after culture, which means that more p53 protein is produced when compared with healthy cells under the same conditions. It must also be stated that western blot experiments were not performed because we mainly wanted to “glimpse” at the mRNA and not the protein level of these patients, and because we had budget issues. It is very likely though, that we will incorporate these methods in our future experiments. 

As stated before, there have been numerous scientific reports showing that a major problem for dialysis patients is chronic inflammation. The major cause of death to this group of patients is for example cardiovascular disease, which can be caused and/or accelerated by chronic inflammation. A state of chronic inflammation is evident by either elevated proinflammatory cytokines (IL-1*β*, IL-6, TNF-*α*, etc.) or elevated acute phase proteins (CRP, albumin, fetuin-A, etc.) [20–23]. While there were not any statistical differences between the serum concentration of IL-6, IL-10, and CRP, the finding that TNF-*α* becomes elevated as the disease progresses is very important and cannot be ignored. It must also be stated that there was no significant statistical correlation between age, sex, smoking, diabetes, hypertension, and so forth and/or any of the serum markers' concentration (data not shown). In this study, we demonstrated strong indications that immune system cells from patients suffering from renal failure have lost their plasticity in regulating the expression of two very important genes which play pivotal roles in the progression of the cell cycle. Is this chronic inflammatory state to be blamed for this dysfunction? This question cannot be answered hereby but this scenario is plausible. TNF-*α* stimulates lymphocytes to proliferate and differentiate when a threat is present in their environment [24, 25]. It is very likely that this constant stimulation causes these cells to become exhausted and senescent. If other laboratories around the world are encouraged by this research to investigate more thoroughly the molecular mechanisms underlying CKD's immune system impairment we will soon uncover these complex molecular pathways. 

Chronic inflammation and immune system dysfunction have been evaluated as major factors in the pathogenesis of CKD and end stage renal disease (dialysis patients), contributing to the high mortality rates observed in these populations. There have been a few studies indicating as well, the emergence of cellular senescence in PBMCs isolated from dialysis patients [17, 26]. The primary thoughts behind this research were to investigate two “classic” tumor suppressor genes in order to see if kidney disease and/or inflammation can influence their expression. Since not so many studies have been published around this issue we consider it as one of the first steps in uncovering how and in what degree renal failure is associated with inflammation and cell cycle. Of course much more studies are required for this goal to be achieved but our results are solid, providing strong indication that something is wrong in the proper progression of the lymphocytes' cell cycle in patients with kidney failure. Our only wish is that our study will provide other scientists with substantial motive in order to trigger them to investigate these molecular pathways through and through. In the present paper, it has been demonstrated that PMBCs from CKD and from dialysis patients fail to regulate the *p53* and *RB* gene transcription as a response to environmental changes. This evidence of molecular senescence in the lymphocytes of renal patients is a matter of further investigation in order to clarify reasons causing the respective immune system impairment. If in the end constant inflammation proves to be the underlying cause under the defense system's dysfunctions, then proper and targeted anti-inflammation therapy must be incorporated in renal patients' therapy.

## Figures and Tables

**Table 1:** Demographic data, inflammation markers, chronic health disorders, and pharmaceutical therapy of the populations included in the study.

		Group	
Patient data	Healthy controls	Chronic kidney disease patients	Dialysis patients
	(*N* = 10)	(*N* = 10)	(*N* = 10)
Age in years (mean/St. Dev.)	49.80/17.59	55.50/10.74	45.60/6.98
Sex (male/female)	8/2	6/4	8/2
GFR mL/min (mean/St. Dev.)	104.40/9.89	37.40/12.35	1.00/0.00
BMI kg/m^2 ^(mean/St. Dev.)	27.58/4.60	26.76/2.90	25.73/3.90
Serum CRP mg/dL, *P* value = 0.445 (mean/St. Dev./Std. error)	0.086/0.187/0.059	0.392/1.026/0.324	0.441/0.493/0.156
Serum IL-6 pg/mL, *P* value = 0.241 (mean/St. Dev./Std. error)	3.180/1.147/0.363	5.070/3.436/1.086	5.070/3.273/1.035
Serum IL-10 pg/mL, *P* value = 0.572 (mean/St. Dev./Std. error)	18.480/6.759/2.137	16.660/5.990/1.894	14.800/9.542/3.180
Serum TNF-*α* pg/mL, *P* value = 0.002 (mean/St. Dev./Std. error)	6.150/0.628/0.198	9.240/3.739/1.182	15.620/7.428/2.349
Smoking*	1	1	2
Diabetes	1	1	1
Hypertension	5	6	6
Atherosclerosis	2	1	6
Heart failure	0	0	2
ACE inhibitors	1	5	1
AT1	1	3	1
CC blockers	1	3	4
Beta blockers	2	2	4
Diuretics	0	2	3
Statins	2	1	1
NSAIDS	0	1	2
Aspirin	0	2	7
Antiplatelets	1	0	6
Phosphate binders	0	1	6
Sevelamer	0	0	6
Vitamin D	0	0	8
Erythropoietin	1	0	10
FE	0	1	8

GFR: glomerular filtration rate calculated with the Cockcroft-Gault formula; BMI: body mass index; ACE: angiotensin-converting enzyme; AT1: sartan; CC: calcium channel; NSAIDS: non steroids anti-inflammatory drugs; FE: iron supplements.

*The numbers in that section of the table and below represent a positive statement. For example one out of ten healthy persons was a smoker while two out of ten dialysis patients were prescribed with NSAIDS.

**Table 2:** Expression of *p53* and *RB* in cultured and noncultured cells.

Pairs analyzed		*p53*			*RB*	
	Difference in expression	Statistical analysis *P* value/standard error range	Difference factor	Difference in expression	Statistical analysis *P* value/standard error range	Difference factor
A before culture—A plain culture	↓	0.000/0.013–0.541	0.131	↓	0.001/0.004–0.553	0.083
A before culture—A culture with LPS	↓	0.000/0.026–0.664	0.190	↓	0.000/0.003–0.577	0.080
A before culture—A culture with CRP	↓	0.000/0.008–0.197	0.048	↓	0.000/0.014–0.265	0.062
A before culture—A culture with LPS-LXA_4_	↓	0.000/0.033–0.903	0.226	↓	0.001/0.053–0.874	0.235
A before culture—B before culture	→	0.056		→	0.260	
A before culture—C before culture	→	0.337		→	0.105	
B before culture—B plain culture	↓	0.001/0.048–0.249	0.176	↓	0.001/0.052–0.622	0.222
B before culture—B culture with LPS	↓	0.000/0.081–0.492	0.202	↓	0.000/0.113–0.614	0.280
B before culture—B culture with CRP	↓	0.000/0.017–0.236	0.075	↓	0.000/0.030–0.408	0.097
B before culture—B culture with LPS-LXA_4_	↓	0.000/0.118–0.807	0.333	↓	0.002/0.157–1.267	0.460
C before culture—C plain culture	→	0.071		→	0.239	
C before culture—C culture with LPS	↓	0.024/0.033–1.608	0.257	→	0.318	
C before culture—C culture with CRP	↓	0.000/0.021–0.250	0.071	↓	0.000/0.060–0.515	0.180
C before culture—C culture with LPS-LXA_4_	↓	0.001/0.054–0.595	0.247	↓	0.050/0.188–1.753	0.500

A: healthy controls; B: CKD patients; C: Dialysis patients; these results are derived from REST software which analyzed the Ct values calculated from the real time PCR experiments.
